# P27 Lymphoma-mimicker of IgG4-related dacryoadenitis and sialadenitis

**DOI:** 10.1093/rap/rkab068.026

**Published:** 2021-10-19

**Authors:** Taha Akhtar

**Affiliations:** Shifa International Hospital, Islamabad, Pakistan. Shifa Medical Center F11, Islamabad, Pakistan

## Abstract

**Case report - Introduction:**

Major salivary gland (sialadenitis) and lacrimal gland (dacryoadenitis) involvement can be a common feature of IgG4-related disease. There can be involvement of lacrimal and parotid gland which was previously called as Mikulicz disease and/or submandibular gland enlargement which was previously called Küttner tumour. These were previously mistakenly considered to be subcategories of Sjogren’s syndrome, but are now classified as IgG4-related disease.

Here we discuss a case report of a patient who presented with bilateral dacryoadenitis and unilateral submandibular gland enlargement which initially was thought to be IgG4-related disease but turned out to be low-grade lymphoma.

**Case report - Case description:**

A 15-year-old boy presented with 6-month history of bilateral eyelid swelling. The swelling was more on the lateral side of the eyelids and was painless. It had come on suddenly over a couple of days. No history of weight loss, dry eyes, dry mouth, joint issues or skin rashes or any other symptoms. Physical examination revealed bilateral ptosis, no visual impairment and systemic examination revealed an enlarged right submandibular gland. He had initially been to a local hospital where he had investigations which included autoimmune screen including ANA, ENA, ANCA, dsDNA, serum ace and complement levels which were all negative. C1 esterase inhibitor was normal. Routine bloods including complete blood count, urea and electrolytes, thyroid stimulating hormone and erythrocyte sedimentation rate were within normal limits. Urine albumin to creatinine ratio was not raised. Hepatitis serology including Hepatitis B & C and HIV was negative. Ultrasound abdomen was unremarkable. CT scan of orbits showed bilateral enlarged lacrimal glands with patchy post contrast enhancement and the glands extending up to insertion of lateral rectus muscle. CT chest some enlarged axillary lymph nodes and nodes in lung query infective etiology. CT abdomen and pelvis was unremarkable. Ultrasound neck showed right submandibular node enlargement with colour doppler showing increased vascularity. Fine needle aspirate of the submandibular gland showed reactive lymphoid hyperplasia. He was given two short courses of steroids and each time the swelling rapidly responded to the steroids but recurred on cessation of the steroids. IgG subset analyses revealed elevated IgG4 levels of 1152mg/dl. The differential here was IgG4-related disease but as there was no clear tissue diagnosis a core biopsy of the right submandibular gland was done. This revealed tissue suspicious of low grade (extranodal marginal zone and mucosa-associated lymphoid tissue [MALT]) lymphoma and excision biopsy was performed for definitive diagnosis.

**Case report - Discussion:**

IgG4-related disease is an immune mediated fibroinflammatory condition which can affect a variety of organs and can present as tumour-like enlargement and/or organ dysfunction. The pathological findings in IgG4-related disease are lymphoplasmocytic infiltrates of IgG4-positive cells along with increased levels of serum IgG4 levels.

Salivary and lacrimal glands can be commonly affected and present as enlargement, which is usually painless and bilateral. The combination of lacrimal gland enlargement with both parotid and submandibular gland enlargement is called IgG4-related Mikulicz disease.

Apart from salivary glands, another commonly affected organ is the pancreas which can present as a pancreatic mass and painless jaundice, sclerosing cholangitis, retroperitoneal fibrosis, aortitis and periaortitis. Less commonly it can affect thyroid, kidney and lungs.

Early recognition, diagnoses and treatment is important due to the fibroinflammatory nature of the disease. Malignancy is always in the differential and should be excluded. Steroids are the mainstay of treatment. If patients experience flare, rituximab can be added.

Diagnosis should be confirmed with biopsy but histopathological findings are never alone diagnostic of IgG4-related disease and should be interpreted with clinical, serological and radiological findings.

**Case report - Key learning points:**

Although this patient had typical presentation of IgG4-related disease with painless enlargement of salivary and lacrimal glands and elevated IgG4 serum levels, biopsy was imperative to get to the diagnoses of low-grade lymphoma and fine needle aspirate was not adequate. As mentioned above, biopsy in IgG4-related disease will confirm the diagnosis provided there are other supporting features (radiological and serological). However, it is imperative for excluding other important diseases like lymphoproliferative disorders.

